# Unveiling the social performance of selected agri-food chains in Costa Rica: the case of green coffee, raw milk and leafy vegetables

**DOI:** 10.1007/s11367-021-01964-4

**Published:** 2021-09-09

**Authors:** Laura Brenes-Peralta, María Fernanda Jiménez-Morales, Rooel Campos-Rodríguez, Matteo Vittuari

**Affiliations:** 1 DISTAL, University of Bologna, Bologna, Italy; 2 Agribusiness School, Tecnológico de Costa Rica, Cartago, Costa Rica

**Keywords:** S-LCA, Agri-food system, Costa Rica, Social impact, Coffee, Vegetable, Raw milk, SAM

## Abstract

**Purpose:**

Several frameworks coincide in the importance of addressing social impacts to ensure sustainability. However, the agri-food sector, regarded as key in sustainable production, still neglects to identify potential social impacts when applying life cycle approaches. This work contributes to understanding the social performance of three agricultural products from a Latin American and Caribbean developing country as Costa Rica while recognising the challenges of Social-Life Cycle Assessment (S-LCA) application in this context.

**Methods:**

S-LCA represents a powerful technique to evaluate the potential social impacts of a product. Three case studies were analysed through S-LCA, using the subcategory assessment method (SAM) to characterise the social impacts and detect hotspots in the production of green coffee, raw milk and leafy vegetables. Primary data was collected through questionnaires to relevant informants and observations. In addition to secondary information, these data and information were used to assess eight impact subcategories for the farmer and worker stakeholder groups and nine subcategories for the local community.

**Results and discussion:**

The main results suggest that the Costa Rican institutional and market frameworks provide an enabling environment for a generally positive social performance in the studied cases. The assessed stakeholders can fulfil basic needs through access to inputs and services and achieve fair-trading conditions. Child labour, forced labour and evidence of environmental or health risks for the surrounding communities were absent. Important efforts to address the delocalisation, migration and child labour were observed, suggesting the potential development of social handprints in further studies. However, the farm production phase, related to farmers and workers, entails hotspots regarding social security and women’s empowerment. Moreover, farmers appear as the most vulnerable group because of their overall social performance.

**Conclusions:**

S-LCA helped identify relevant areas of intervention in the context of these particular case studies; however, further research and capacity building are recommended to tackle the detected challenges, both in the agri-food chains and in the use of S-LCA. Furthermore, these findings can aid in future decision and policy-making to improve and safeguard the positive social performance observed in the studied products.

**Supplementary information:**

The online version contains supplementary material available at 10.1007/s11367-021-01964-4.

## Introduction

Sustainable development has been part of international and national policies for several decades. Years after the release of its formal definition by the Brundtland Commission’s report of 1987 (Brundtland, 1987), our global community presented a set of strategic actions known as the Millennium Development Goals. The most current version of actions is contained in the Sustainable Development Goals (SDGs), aiming to achieve such development by balancing the environmental, economic and social dimensions (Manik et al. 2013). However, many efforts and studies usually focus on the environmental challenges (Fauzi et al. 2019), neglecting the social perspective, even when human well-being is a crucial aspect of sustainable growth (Mani et al. 2016). Moreover, the evaluation of social sustainability is intricate since it involves different stakeholders and areas of attention entailed in modern and complex food supply chains (García-Herrero et al. 2019). Latin America and the Caribbean (LAC) has become a relevant region to study sustainability since it is responsible for 14% of the world’s agri-food production and 23% of global exports. However, investment in the LAC agri-food sector is still lower than the OECD countries and global averages, potentially causing constraints in food security and nutrition, health, poverty, traditional livelihoods and migration (OCDE/FAO 2019). Consequently, such social limitations call for actions to improve policies, investment and research.

SDG 12 for Sustainable Production and Consumption demands a systemic change, decoupling economic growth from environmental degradation in all phases of the life cycle of products. Methods have been developed to address this goal. Among those, the life cycle thinking (LCT) approach has been recognized as powerful to examine sustainability in integrated manners and not solely from the environmental standpoint (Salla and Castellani 2019; Manik et al. 2013; Parent et al. 2013). Particularly, social life cycle assessment (S-LCA) is defined as a technique that evaluates the social impacts in relation to a stakeholder over the life cycle of a product (UNEP 2020). In addition, further application of S-LCA allows observing the social footprints (or impacts) and handprints, allowing to see also the positive impacts (Norris et al. 2019). Most S-LCA available case studies in the literature are focused on the manufacturing or the agricultural sector, and almost half of them have been implemented in developing countries. Even when increasing publications ground S-LCA as the main methodology to assess social sustainability, there are still gaps in its implementation. Some of these gaps relate to the inventory methods and analysis, the definition of the goal and scope, the scales and type of assessment, the definition of acceptable and non-acceptable outcomes and its geographical relativity (Sureau et al. 2020; Tokede and Traverso 2020; Fauzi et al. 2019; Lucchetti et al. 2018; Petti et al. 2018a, b).

S-LCA can become a valuable tool to improve social performance in parts of the world such as the LAC Region, especially when such studies are still scarce (Cornejo and Orner 2019; Du et al. 2019a, b; Du et al. 2019a, b). The results obtained from this type of research are crucial both for improved inclusiveness within the agri-food sector, and to remain a key player in the global food markets (OCDE/FAO 2019), even after the COVID-19 pandemic. Therefore, the purpose of this paper is to aid related stakeholders, such as decision-makers in each selected agri-food chain and policy-makers, in understanding the social performance of their agricultural products from Costa Rica, a developing country from LAC. Our paper collects three case studies: green coffee, raw milk and leafy vegetable production. It uses a Reference Scale S-LCA approach, aggregated under a theme for each assessed stakeholder, with the support of the subcategory assessment method (SAM). The interpretation of the social performance, and the detection of hotspots and possible trade-offs, could potentially guide policy-making processes and strategic actions for each sector, also showcasing the prospects of S-LCA application in this particular context.

## Materials and methods

### Case studies description

Costa Rica is a democratic country located in the Central area of LAC. It has had steady economic growth, one of the lowest poverty rate in the region, upper-middle-income nation and high sustainability indicators. Unfortunately, current conditions related to the COVID-19 pandemic are pressing into increased inequality (World Bank 2020). The agri-food sector generates 12.3% of the jobs in the country, supports 5.2% of the national economy and 45.7% of the exports (SEPSA 2016). Almost half of the national territory is dedicated to agriculture (47.1%), and a similar proportion is for conservation, with increased urbanisation processes in concentrated areas (PNUD 2019). The activities from the case studies (Table 1) were selected due to their relative contribution to the national economy and food security, their local relevance and the relative absence of these primary products in prior S-LCA studies (Huertas-Valdivia et al. 2020). For instance, the dairy sector farms represent 28.5% of the national agricultural coverage; coffee production represents 24.3% and vegetables 4.8% (INEC 2018). A more specific description of the cases is also provided in the “Results and discussion” section.

**Table 1:** Description of S-LCA case studies

Crop	Green Coffee	Raw milk	Leafy vegetables
**Farms**	6	4	3
**Functional unit**	1 kg of green coffee	1 kg of raw milk	1 kg of lettuce
**System boundaries**	Cradle to farm gate	Cradle to farm gate	Cradle to market entry gate
**Considered stakeholders**	Workers, local community and value chain actors (farmers)		
**Interviewed key informants**	12	11	5
**Key informants**	The group included farmers, cooperative agents and institutional actors	The group included farmers, workers, sectoral representatives and institutional actors	The group included farmers, workers, members of the farmers’ association and institutional actors

### Social-life cycle assessment

The first guidelines to perform S-LCA were published in 2009 by UNEP and SETAC. Evaluations using them have increased in the past decade (Huertas-Valdivia et al. 2020; Fauzi et al. 2019), contributing to further developments and more specific delimitations, as in the S-LCA Guidelines of 2020 (UNEP 2020). S-LCA informs of the performance of a product or service regarding potential social impacts or risks, footprint or handprints providing support for decision-making and discussion to advance towards compliant conditions for human rights and, in general, well-being. It does not provide particular solutions nor is defined by a standardised method but follows the same steps from the (Environmental) LCA facilitating the detection of hotspots and trade-offs (UNEP 2020).

There are several approaches to address a S-LCA. The interpretivist paradigm (Iofrida et al, 2017; Russo-Garrido et al. 2018) was followed in this case. Type I categories were used instead of type II categories that model the results through causal links. Impact categories were evaluated through reference points, and after being scored, they were aggregated under themes for each assessed stakeholder. This latter step is considered relevant for actual S-LCA assessment instead of simple scoring against pre-established metrics (laws, standards, practices).

#### Goal and scope

**Fig. 1:**
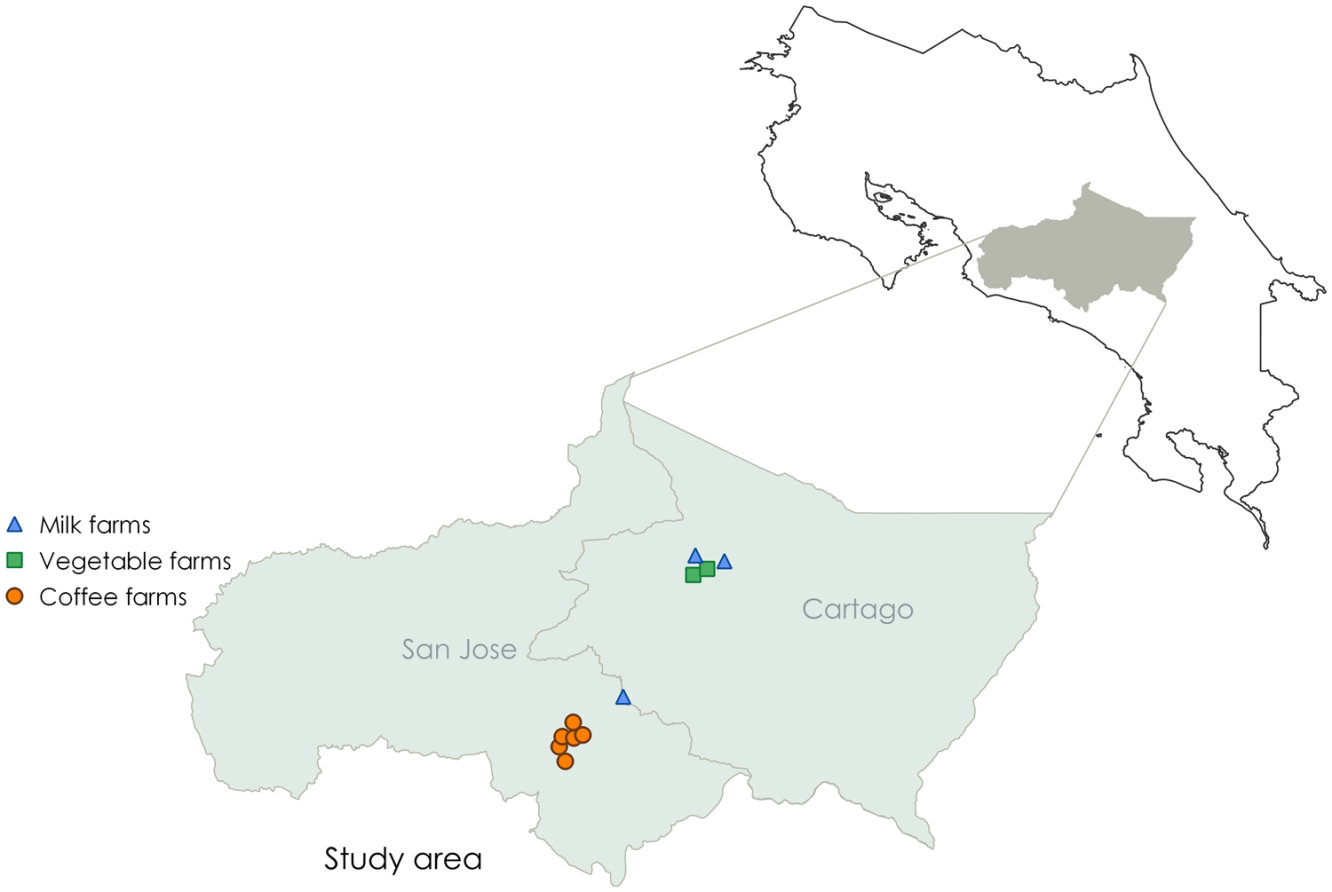
Location of the three case studies in Costa Rica

**Fig. 2:**
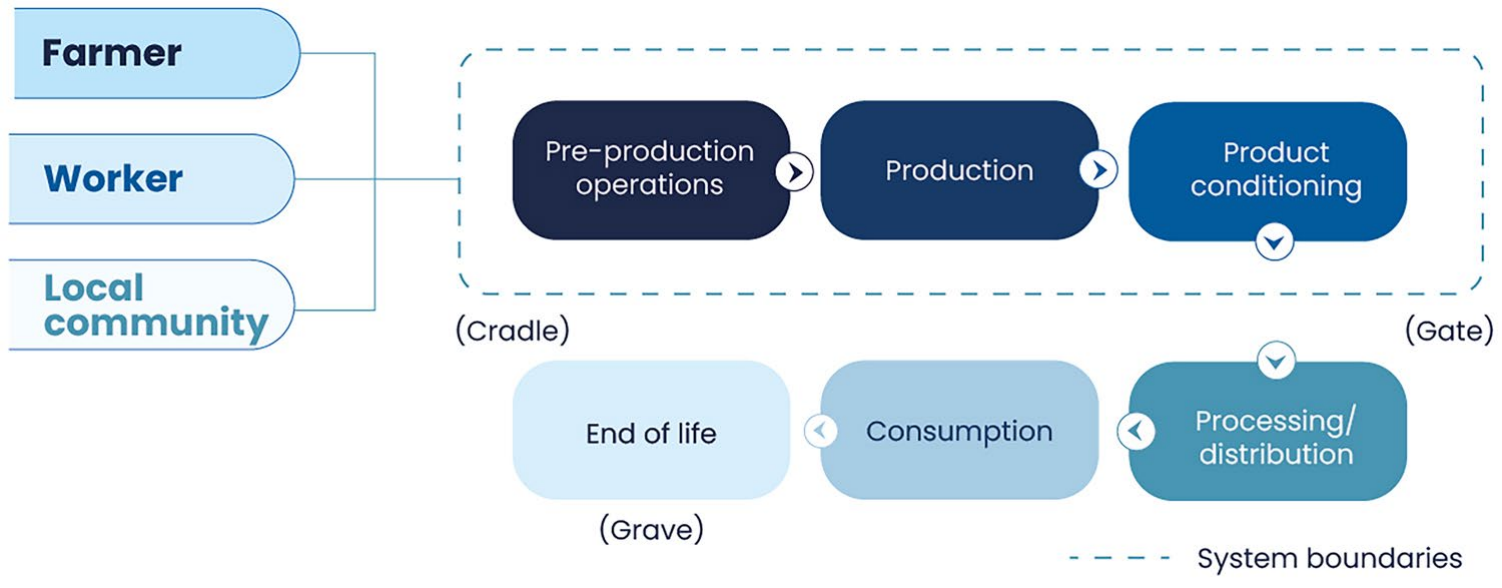
System boundaries and stakeholders of the S-LCA of the case studies

The study had the goal of assessing the social performance and detecting the main hotspots in the production of 1 functional unit (FU) of green coffee, raw milk and leafy vegetables, with farms located in the central zone of Costa Rica (Fig. 1). The system boundaries considered life cycle stages from cradle to gate (Table 1; Fig. 2). The detection of opportunities, good practices and improvement needs in the studied subsectors intends for farmers, value chain actors and other stakeholders to consider the outcomes of this study in their decision-making, strategies and policy processes. In this way, actions could be aligned to overcome constraints and empower strengths towards improved social performance in the future.

This research assessed three of the stakeholders referred by the UNEP Guidelines (2020) (Fig. 2). The stakeholder group of workers from the 2020 Guidelines was modified, placing particular emphasis on farmers, similarly to the small-entrepreneurs stakeholder category of the S-LCA methodology by Goedkoop et al. (2018). This was due to the fact that most agricultural activities in LAC, in Costa Rica and particularly in the three case studies, are developed by medium or small family farmers (OECD 2017), considered by authors as meritorious for a separate group, sometimes invisibilized in value-chain actors or worker categories.

#### Inventory analysis

This phase resulted from collecting data through focus groups and applied questionnaires to a non-representative sample of farmers, workers and sector experts. Due to the novel application of S-LCA in this country, a case-study approach was followed. Interviewees were approached based on their relation to each case and their willingness to participate. On-site observations and primary documentation when available were also considered, as well as secondary national or sectoral information, related to national or international legislation on labour rights (MTSS 2018; 2019), including child labour (FAO 2020; PANI 2020) and migrant working permits and social security coverage (MAG 2020; ICAFE 2019; Loría-Bolaños 2012); national and agriculture-sector statistics and analysis (INEC, 2015; 2018; MTSS/CSO 2018; OECD 2017; SEPSA 2016; INDER 2016; de la Garza Toledo 2001) and subsector statistics and reports (ICAFE 2020a, b; CoopeTarrazú 2019; Coto-Keith 2019; CNPL 2019; 2018; 2017; MEIC 2017; Barboza-Arias 2016). Labour costs were also inventoried for each life cycle stage of the studied products to later consider in the materiality assessment as part of the interpretation phase of the S-LCA.

#### Impact assessment

This study used the Reference Scale S-LCIA approach (UNEP 2020) to provide a social performance assessment through a value scale. The impact assessment phase consisted of the application of SAM as a characterisation method, expressing a score based on a four-level reference scale for the evaluated system (D’Eusanio et al. 2018; Petti et al. 2018a, b; Sanchez-Ramirez et al. 2014):

- Level A (value of 4) is obtained when the system or the organisation responsible for the assessed product shows a proactive attitude, surpassing Basic Requirements (BR)
- Level B (value of 3) indicates the fulfilment of the BRs
- Level C (value of 2) is assigned when BRs are not met, similar to peers or the local context
- Level D (value of 1) is assigned when BRs are not fulfilled, while the sector or context usually does or is close to compliance

The BRs were established according to national legislation (aligned with international dispositions) and context conditions and practices. The study included 114 indicators concerning the impact subcategories (Table 2), based on the Methodological Sheets for Subcategories in S-LCA (UNEP/SETAC 2013) (indicators can be seen in the Supplementary material section). Answers from the different interviewees were recorded in Microsoft Excel ® spreadsheets to be analysed with basic descriptive analysis, and since results were presented as ordinal data, the median was used (Harpe 2015) together with triangulation to score the potential impacts by subcategory.

**Table 2:** Stakeholder groups and impact subcategories

Stakeholder	Source of evidence	Impact subcategory
Farmers (value chain actors)	Questionnaire, interviews, non-participatory observation, secondary data	Meeting basic needs Access to services and inputs Women´s empowerment, inclusion and no discrimination practices Child labour Health and safety Land rights Corporate responsibility Fair competition
Workers	Questionnaire, interviews, non-participatory observation, secondary data	Freedom of association and collective bargaining Child labour Fair salary Hours of work Forced labour Equal opportunities/no-discrimination Health and safety Social benefits/social security
Local community	Questionnaire, interviews, non-participatory observation, secondary data	Delocalization and migration Community engagement Cultural heritage Respect of indigenous rights Local employment Access to immaterial resources Access to material resources Safe and healthy living conditions Secure living conditions

Most impact subcategories are based on UNEP (2020), except the ones for farmers, considered a particular value chain actor based on Goedkoop et al. (2018)

Moreover, an overall social performance assessment (OSP) was provided for each stakeholder of the studied cases, within values from 0 to 100 (formula 1) and assuming equal weight among impact subcategories, which helped detect the most vulnerable stakeholders.

1 $$OSP=\left(\frac{TPP}{OP}\right)\ast100$$

TPP is the total possible points to be obtained for each stakeholder, and OP is the actual obtained points during the assessment.

Table 3 summarises the contribution of the hours of work to each process occurring in each agri-food chain according to the applied data collection processes. Further discussion was offered; and hotspots, and trade-offs were detected.

**Table 3:** Working hours contribution per life cycle stage on each case

Case	Life cycle stage	Cost (US$/kg)^a^	% of contribution
**Green coffee**	Pre-production	0.01	0.39
	Production until harvest	1.52	96.25
	Product conditioning	0.05	3.36
**Raw milk**	Pre-production	0.09	36.96
	Production until harvest	0.11	47.43
	Product conditioning	0.04	15.61
**Leafy vegetables**	Pre-production	0.45	51.72
	Production until harvest	0.33	37.93
	Product conditioning	0.09	10.34

^a^Kilograms of the defined functional unit for each case

#### Interpretation

Finally, this phase was built in iteration with the prior phases to check for completeness and consistency with the goal and scope, as well as the data for the inventory and impact assessment. The authors also offered a discussion on the most relevant subcategories, stakeholders and lifecycle stages for the context of the case studies, as well as the observed limitations and useful applications of S-LCA in the studied agricultural context, as a complement to the materiality assessment.

## Results

### Green coffee

This case study, consisting of six small coffee farms located in the Tarrazú canton in the ‘Los Santos Region’, included conventional shaded coffee production systems that use coffee brush compost and bioinputs (CoopeTarrazú 2019). Farmers send the harvested coffee beans to be processed and commercialized through a local cooperative named Coopetarrazú. Most of the tasks are manual, with an increased number of workers during harvest season (ICAFE 2020a, b). The human workforce is distributed as follows in each life cycle stage to produce 1 kg of green coffee: 0.4% is used in the pre-production stage, 96.2% in production at the farm level and until harvest and 3.4% in the conditioning stage (milling).

**Fig. 3:**
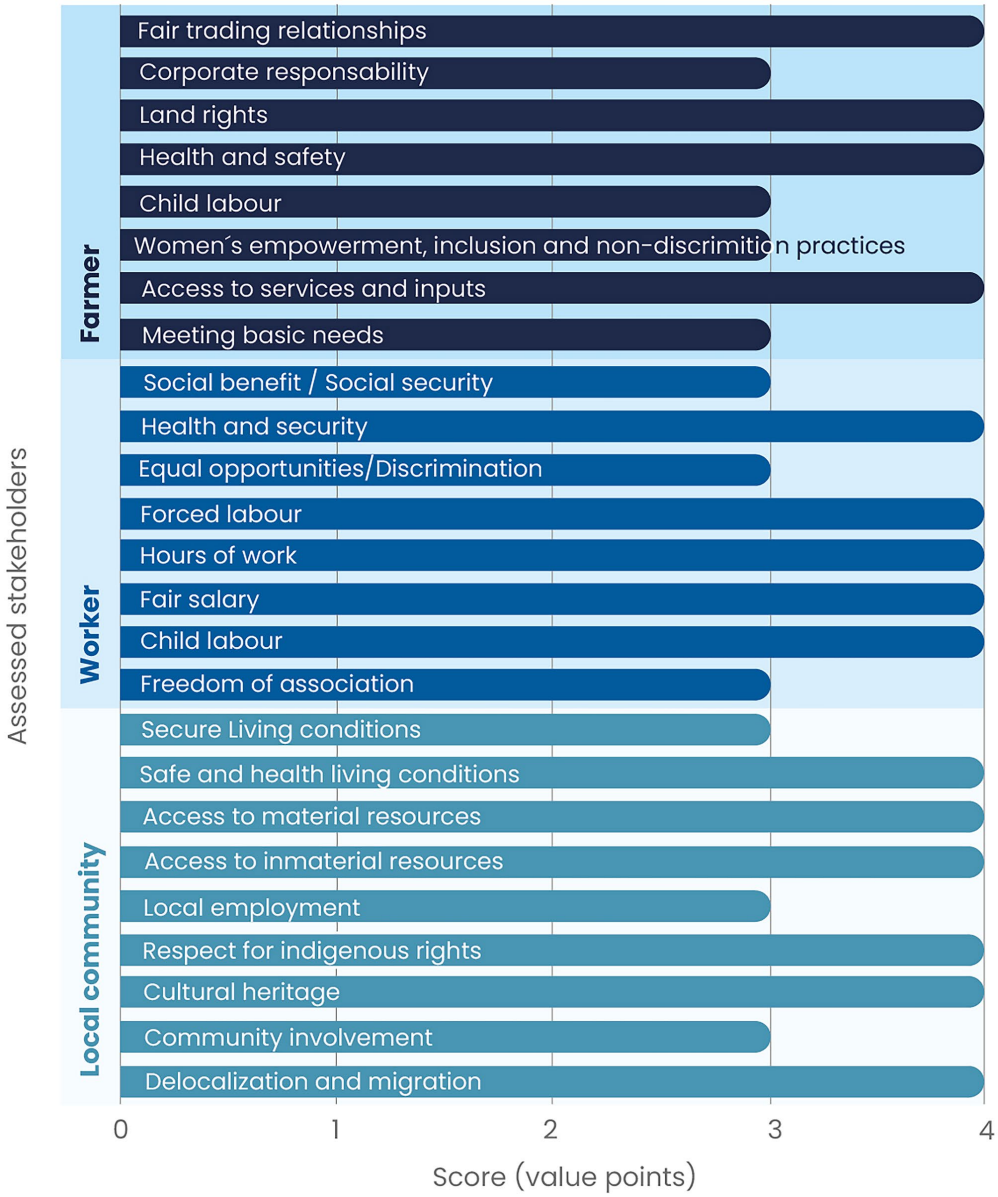
Subcategory assessment results in the green coffee case study

The farmers were the first stakeholders to be assessed in this case. The subcategories of *access to services and inputs*, *health and safety land rights,* and *fair-trading conditions* obtained a level A score with a value of 4 points, while the remaining social impact subcategories obtained level B scores, meaning they complied with BRs but did not surpass them. The assessment for the workers’ stakeholder group is in general positive since five subcategories obtained a proactive performance score (level A, 4 points), namely *child labour*, *fair salary*, *hours of work*, *forced labour,* and *health and security*; the remaining three subcategories obtained a level B score. A third assessed stakeholder group was the local community, where the following subcategories obtained level A scores: *delocalization and migration,* *cultural heritage*, *respect to indigenous rights*, *access to material resources*, *access to immaterial resources,* and *safe and healthy living conditions*; in contrast to *community involvement*, *local employment* and *secure living conditions* that obtained level B (3 points) scores (Fig. 3).

### Raw milk

**Fig. 4:**
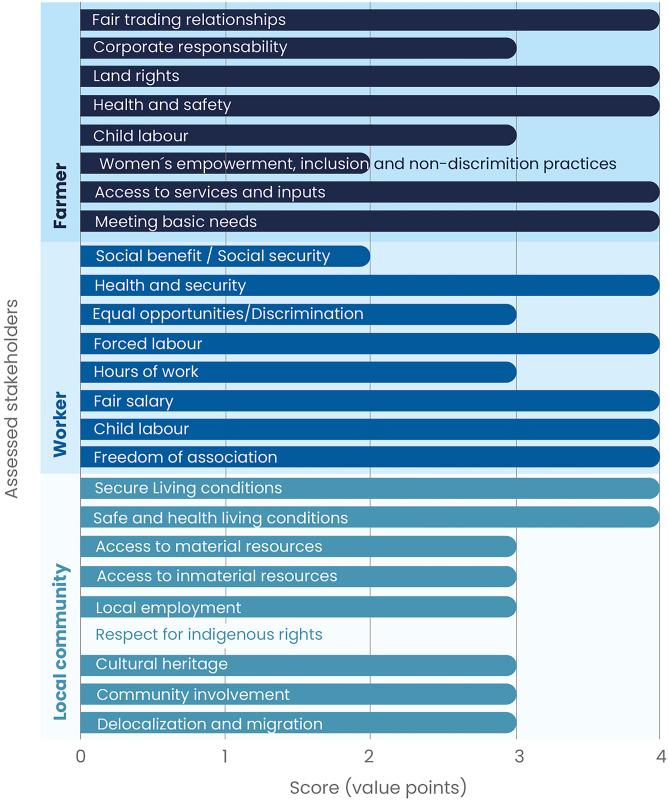
Subcategory assessment results in the raw milk case study

This case study included specialised high-land dairy farms in the Cantons of Alvarado, Oreamuno and Tarrazú, belonging to two of the largest milk-producing provinces in Costa Rica (Arndt et al. 2020). These are characterised as commercial farms that turn their milk output to their own cooperative for industrialisation, with high-quality milk production, dairy breeds and high milk yield–oriented feeding strategies. According to farmers, the involved human force dedicated to the life cycle stages of the production of 1 kg of raw milk corresponds to 36.96% in the pre-production stage (calf, heifer and cow growing, pastures and feed production), 47.43% in the milk production and milking operation and less than 15.61% in the milk conditioning for further processing stages. Figure 4 presents the social impact subcategories assessed for the three stakeholder groups of the study. The following subcategories from the farmers’ stakeholder group obtained level A scores: *meeting basic needs*, *access to services and inputs*, *health and safety*, *land rights,* and *fair-trade relationships*; c*hild labour* and *corporate responsibility* subcategories obtained a B score, and *women´s empowerment*, *inclusion and non-discrimination practices* scored at C level, mostly due to the absence of female workers or farmers during the interviews and observation processes. Workers' impact subcategories of *freedom of association*, *child labour*, *fair salary*, *forced labour,* and *health and safety* obtained a level A; *hours of work* and *equal opportunities* subcategories ranked at the B level, and *social benefits* and *security* obtained a C level score. The last stakeholder group assessment of social impact subcategories in the raw milk production case was for the local community, where subcategories related to *safe and healthy living conditions* and *secure living conditions* in regard to the local community stakeholder group obtained a level A score, and the remaining a B score, except indigenous rights which was not applicable to the case.

### Leafy vegetables

**Fig. 5:**
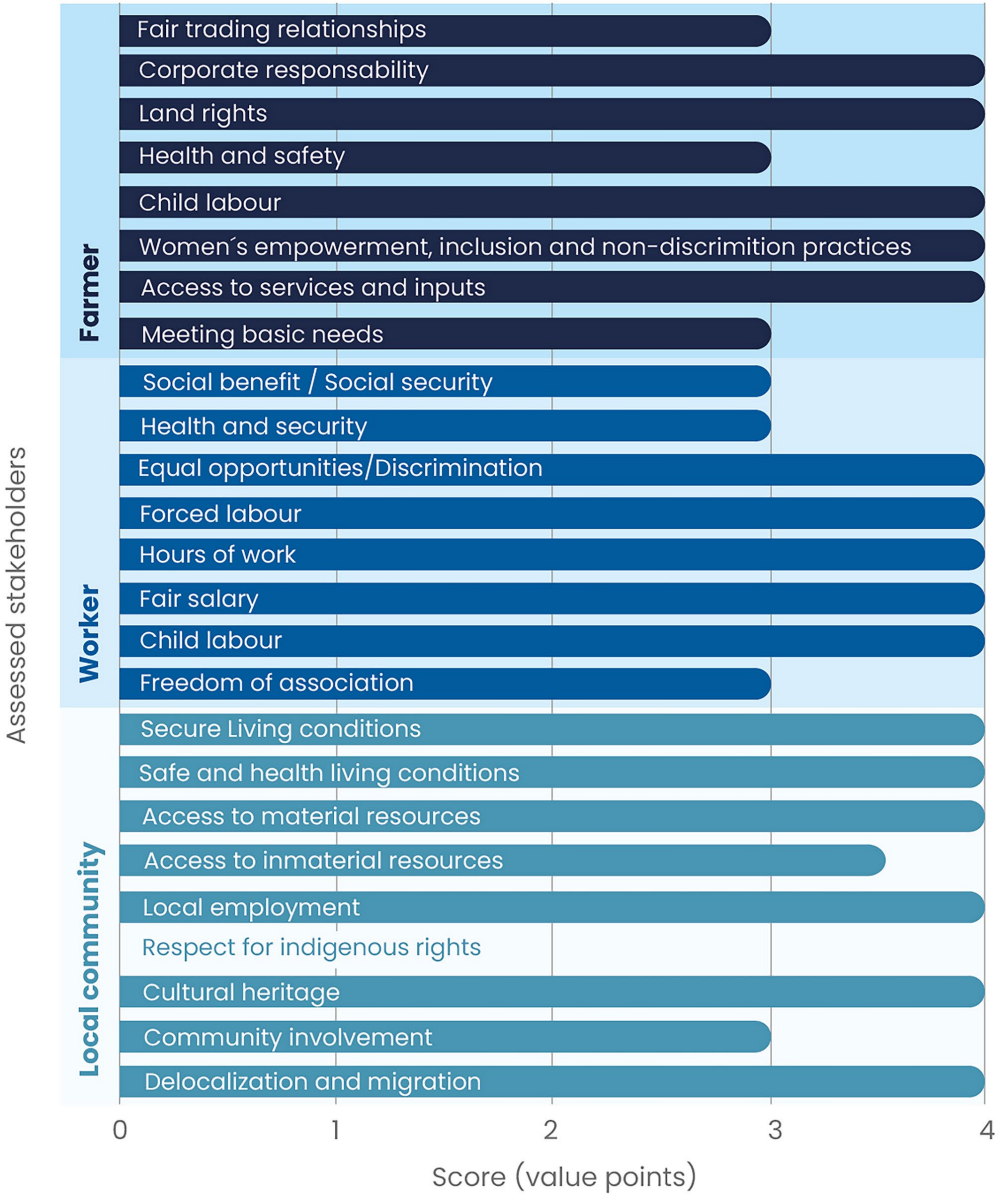
Subcategory assessment results in the leafy vegetable case study

The leafy vegetable case study was built on three farms that operate within a local organic farmer association in the northern part of the province of Cartago called APROZONOC. They qualify as small family farms with improved environmental sustainability performance according to the ‘Bandera Azul’ award and the Primus Lab Organic certification scheme (PBAE 2017) (Pacheco-Rodríguez et al. 2017). Their product is commercialized in farmers’ markets during the weekends or through personalized delivery during weekdays. Farmers indicate that 51.72% of the human force is used in the pre-production phase, 37.93% in the production phase and 10.34% in the conditioning phase. The following social impact subcategories were assessed as A level or proactive (value of 4 points) for the farmers’ stakeholder group: *access to services and inputs*; *women´s empowerment*, *inclusion and non-discrimination practices*; *child labour*; *land rights and corporate responsibility*. Subcategories regarding *meeting basic needs*, *health and safety* and *fair-trade conditions* were assessed at B level. Regarding the workers’ stakeholder group, indicators for the subcategories of *child labour*, *fair salary hours of work*, *forced labour and equal opportunities and non-discrimination* obtained a score of A. In contrast, *freedom of association*, *health and security*, *social benefits* and *social security* obtained a level B score. Finally, the assessment of social impact categories for the local community stakeholder group considered most of them to be proactive (level A). Two categories complied with BRs (level B), and the one regarding indigenous rights does not apply since indigenous peoples are not present in this activity (Fig. 5).

**Fig. 6:**
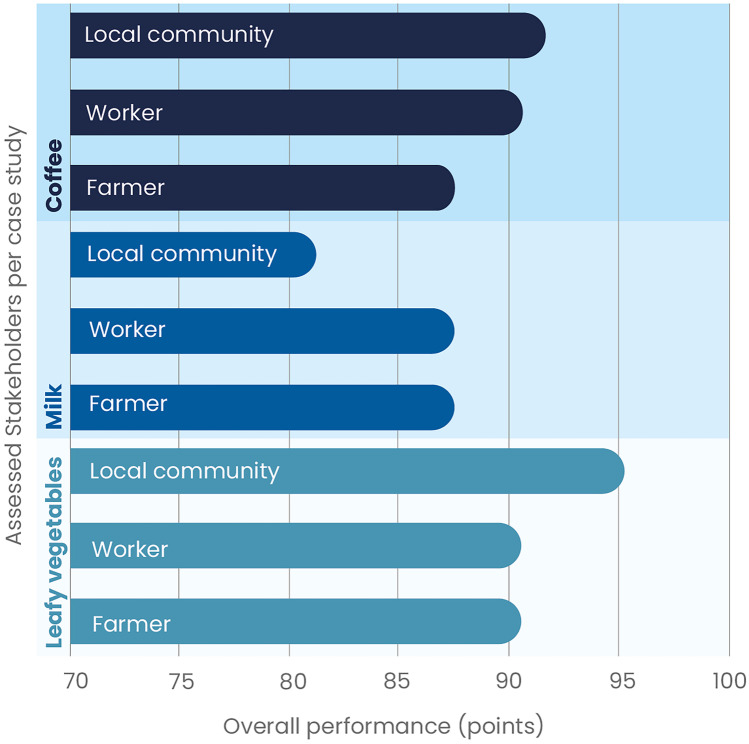
Stakeholder performance per each case study

A summary of the aggregated assessment from each of the studied cases (Fig. 6) presents the overall performance per stakeholder.

## Discussion

### Social performance of the studied products, general traits and strengths

The three case studies present a satisfactory social performance as a response to a generally enabling environment provided by a robust social and institutional national system, and the decisions and efforts from the productive sector to avoid negative impacts on stakeholders. These outcomes can be traced to the existence of work legislation and policies that foster decent work hours, minimum wages, avoidance of child and forced labour, freedom of association, collective bargaining and the will to comply beyond the minimum in many cases. There are also public and accessible services for extension and training, public transportation, electricity and potable water supply in the areas of the study. In addition, commercial conditions allow the provision of a variety of inputs, input-suppliers, telephone, credit and insurance services, both from public and private operators (Loría-Bolaños 2012; INDER 2016; OECD 2017; World Bank 2020).

The environment and health-related legislation, practices and standards in the country and these sectors, together with the companionship of academic, research and training institutions (OECD 2017), have also played a key role in preventing social risks and in fostering safe and healthy living conditions (in the farms and from the farms towards the community). Despite agriculture activities being in the top three positions for work-related injuries (MTSS/CSO 2018), the cases did not present fatalities and recorded low accident and illnesses rates related to farm chores. Stakeholders agreed that workers are always provided with safety equipment and are also encouraged to work carefully to avoid accidents. These later conditions act as an alternate mechanism to explicit policies or safety departments, as they are not mandatory due to the number of workers per farm (less than 20 persons year^−1^) (MTTS 2018).

Waste, environmental and health regulations are established at the constitutional level in Costa Rica (Rodríguez-Becerra and Espinoza 2002), nudging the studied activities to shift from top-polluting conditions to improved health and working circumstances. Currently, many waste management and biocircular economy examples in the country (MICITT 2020) are born from these agriculture subsectors. Moreover, organic farms operating in the country, and particularly the ones from the leafy vegetables case study, would not only comply with pest-control residues monitoring, but they also show a high commitment to comply with other voluntary standards (PBAE 2017; Pacheco-Rodríguez et al. 2017). This suggests lower risks of pollution and health hazards due to chemical fertilisers or pest-control inputs in the produce they sell and their surroundings. Similar to a perception study developed by Racines, Isaías-Acuna and Varela (2021), organic vegetables are perceived positively based on elements related to health, protection to the land and sensitivity to farmers.

Two of the cases operate under cooperative schemes (coffee and dairy), which support farmers in fair trade, training and services opportunities and improved health, environmental and safety circumstances (Barboza-Arias 2016). The difference from commonly independent productive units in contrast to organised cooperative ones also suggests the development of alliances within the value chain actors and other stakeholders (CNPL 2017; ICAFE 2020b). It also seems to set an enabling condition to achieve certifications (CoopeTarrazú 2019) and comply with regulations that support fairer quality standards and product technical categorisations at the national or regional level (MEIC 2017; CNPL 2019). Farmers grouped in this model get involved in their communities as well, whether they directly organise the activities or support the main organisers. Moreover, the business model usually grants common benefits for the cooperative members and the surroundings. For example, Coopetarrazú awarded over US$90,000/year to the local community in 2019 through different activities and infrastructure (CoopeTarrazú 2019). However, this was not the case for the leafy vegetables, since even when associations, national programmes and organisations were identified, farmers regretted the absence of real support for transparent and fair commercialisation of organic products. The latter was due to policies that have not been reinforced to consistently request organic production evidence in certain local markets or supply chains.

Additional and extraordinary measures have been observed in the case of coffee production. On the one hand, respect for indigenous people from the Ngäbe-Buglé community (Morales-Gamboa, Lobo-Montoya, & Jiménez-Herrera, 2014) who come for the harvest season is present in regard to health insurance and migration status. On the other, a substantial investment has been made in a social project called ‘Casas de la Alegría’. This initiative aims to provide children and minors that move with their parents during the harvest season, indigenous or not, with proper attention while the parents are on the farms (UNICEF/IMAS 2019; CoopeTarrazú 2019). Not all coffee production regions in Costa Rica or Mesoamerica present this particular asset, expressing a concrete action where farmers, the cooperative managers and the institutions have created an evident positive result in child labour indicators.

### Social hotspots and trade-offs

The percentual distribution of human workforce per each impact category suggests that operations occurring at the farm level entail the highest potential risk of social impacts in the life cycle of these products. For example, the green coffee case and the raw milk cases find the highest contribution at the production stage; the leafy vegetable case has higher contributions at the preproduction phase. Consequently, the farm level is a hotspot to be addressed whenever interventions are considered, both by productive actors and by policy-makers. If this hotspot was not properly addressed, vulnerabilities could become evident in some of the impact subcategories or indicators that showed slightly poorer or more challenging performances. Therefore, the following can be considered entry points to conduct future diagnoses or interventions.

It was observed that farmers, their families and workers could access basic living conditions thanks to coffee, milk or vegetable production. However, in some cases, farmers claimed to be unable to surpass basic needs. Therefore, even when they situate above the line of poverty (INEC 2018), rural families tend to be at risk of food insecurity (Intini et al. 2019). This aspect is most certainly increased by the crisis caused by the COVID-19 pandemic that started in 2020.

Social security was one of the topics related to both farmers and workers: health coverage is spread, public and accessible country-wise; however, farmers consider the affiliation to this system to be expensive. Therefore, in some cases, the workers resort to social security coverage paid by themselves. This is possible under the national legislation for freelance workers with non-permanent attachment to an employer; however, it was not possible to determine if this was the situation at all times in these three case studies. This situation is quite relevant from two perspectives, as it becomes the most pressing trade-off observed in this assessment. On the one hand, most health and social security infractions in Costa Rica related to the enrolment and payment of social security and salary records (MTSS/CSO 2018). Coincidently, farmers, who stated that the cost of social security is high for them, might not enrol their workers since it might affect their income and profit. In parallel, if income is severely affected, it could cause a reduction of hired workforce and job generation.

On the other hand, the agriculture sector is the fourth highest private contributor to the Costa Rican social security system (MTSS/CSO, 2018); thus, the contraction of the contribution could severely impact the sustainability of the system. Outcomes of studies like ours can aid in making visible these trade-offs and constraints, presenting the need to address these risks not solely from the farmer or the law enforcer perspective but throughout the value chain. Buyers and consumers might not be aware of these issues, and visualizing the importance of fair trade and price if they expect that the goods they purchase are produced sustainably and fairly should take these matters into consideration.

Women’s empowerment is another aspect that still requires close observation, as most decision-making processes from the studied farms still lack the full and equitable involvement of women. National statistics indicate 15.6% of farmers are women, and 37% of the occupied national workforce is female (INEC 2015; 2018). Despite when these thresholds were closely met in the coffee and leafy vegetable cases, the raw milk case lacked female participation, according to observations and the participants farms did not hire female workers nor had women in decision-making positions. Even when interviewees of all cases expressed the absence of discrimination, certain aspects related to gender, sexual orientation and diversity were not fully discussed or approached.

Local employment is another relevant aspect to address in the Costa Rican agriculture sector, regarded as a second socio-economic *vs* productive trade-off. This is evident in coffee production since even when there might be an interest to hire local workers, most farm operations are dependent on migrant workforce (Loría-Bolaños 2012). The condition born from this indicator represents a high risk for other economic and productive indicators, and migratory conditions, which could limit the workers’ access to social security and health coverage. The awareness of the situation has motivated a set of alternatives that entail the chance to normalize or obtain temporary work permits for immigrants to be subjected to social security and verified decent working conditions (MAG 2020).

Finally, even when not significantly affecting the overall score of subcategories, hours of work, fair salary and freedom of association shall be monitored. Documentation to state the working relations, payments and hours of work within the farm operation is not always common. Few farms evidenced some form of information system (notebook, bank deposits or computer information systems) to keep track of their working relations, payment conditions and incidents. Many working contracts were referred to as verbal, and few farms (only from the raw milk case) indicated to provide payment slips or made bank deposits, which also creates evidence of the salary payment. This challenge is already pointed in national statistics where the absence of payslips is one of the most common infractions (MTSS/CSO 2018), and even though the Costa Rican legislation allows verbal contracts when the working relationship is of less than 3 months (MTSS 2018), many long-term relations are not documented. Such conditions can create difficulties in future disputes and affect social accountability. In addition, some productive sectors in Costa Rica have had harsh encounters with the operation of worker unions; therefore, there is a worn-out image towards this type of worker association (de la Garza Toledo 2001). Despite the assessment of the subcategory that suggested there was freedom of association, there is no encouragement to formally establish certain union typologies in the farms of the case studies. Indirectly, this could mean working relations are mostly harmonious for the studied cases (as expressed by workers), but on the other hand, if conflicts arrive, workers have little chance to collectively bargain for their conditions.

### Considerations for social handprints

Although S-LCA baseline studies are not available in the context of this study, significant improvements from the past two decades separate certain traits of these case studies from the business-as-usual (BAU) agricultural production in developing countries. Clear examples like the ones presented in previous paragraphs can aid in the future to perform social handprints. An increased interest is currently seen in literature and the UNEP Guidelines to report the footprints and the positive impacts and benefits that can occur when interventions are planned in a business or sector. Those interventions can produce changes beyond the scope of the footprint and throughout the supply chain.

**Fig. 7:**
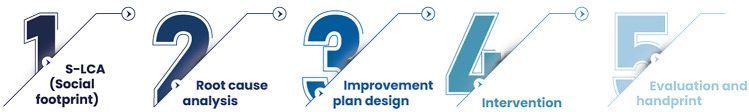
Suggested steps for social handprint in agri-food case studies

Findings from the present S-LCA suggest the first steps for further defining the social handprint framework for these agri-food activities in Costa Rica. Considerations for social handprinting can build from already established frameworks (Norris et al. 2019) and followed paths at the national level that have resulted in proactive performances of the case studies. Taking the green coffee case and how child labour was addressed as an example, a step-wise framework can be suggested. In this case, the footprint can be provided by social assessments (such as S-LCA), allowing the detection of child labour problems. When analysing the causes, different alternatives appear. Then, a plan is designed to improve the situation and tackle the observed footprint or risk to fatherly intervene. As a result of the evaluation of the intervention, a proactive performance in the child labour category was observed. When carefully accounting for baseline conditions and metrics, a handprint can be built (Fig. 7).

The framework can be used in several situations; therefore, an example for further development is presented ahead. The current S-LCA and the footprint outputs of this research suggest social security is one of the biggest contains both for farmers and workers, potentially affecting the contribution to the national system at the farm level (hotspot). Such condition can be translated into working hours under the risk of low social security coverage to provide a particular metric (step 1). When analysing the situation, research and stakeholders can provide insights in regard to the cost of the social coverage and the effect that farmers perceive in their profit (step 2). Therefore, the next step should be dedicated to design a plan for an intervention (step 3), for example, alternatives that can aid in the costing structure or special fees for a specific type of sectors. Then, the intervention would be executed (step 4), and while recording the changes in the metrics outcomes, a new evaluation and handprint calculation (step 5) can be obtained. In this step, the difference between the baseline and the decreased number of working hours at risk, or perhaps an increased number of workers since the farms economy allows for expansion, could evidence the reduction of the negative situation. Moreover, other stakeholders can benefit, such as family members of farmers now covered by social security who can access better health care conditions.

### Prospects of S-LCA in the Costa Rican and LAC context

Strict comparison with other S-LCA studies was not considered feasible due to the case-study nature of our research and differences in functional units, system boundaries, context and products (Tokede and Traverso 2020). However, similar traits within our studies and others suggest further attention and policy efforts are required in particular social areas in agriculture-based products. For example, impact subcategories regarding the promotion of social security, local employment, delocalisation, migration and transparency are hotspots in the honey production (D’Eusanio et al. 2018). Worker-related subcategories achieved basic requirements level but not proactive in the ‘*Cuore di Bue*’ tomato S-LCA by Petti and authors (2018a). Other studies also suggest that subcategories related to farmers (workday length, workload, and professional development, among others) were compliant with the local context regulations in the Canadian dairy sector (Revéret et al. 2015).

While conducting the assessment, the researchers encountered challenges for data collection since record-keeping at these small-scale operations is not always common. This is a challenge to develop research and S-LCA method application, and for transparency and monitoring of the activities themselves. Data based on observations and questionnaires, which would entail testimonies of the interviewees as inputs, were required, entailing possible subjectivity in the study. This latter is considered to be one of the weak aspects of the assessment itself. Even when expressions of different stakeholders and key informants were taken into consideration and contrasted with secondary data, questionnaires and interpretation of the answers are a delicate matter regarding representativeness and possible standardisation to avoid biases. The researchers also perceived that some questions caused uneasiness to some parties, producing a lack of answers or the preference of omission of those in the data collection process. Moreover, there is still limited understanding of the S-LCA method within some informants.

LCT is not widely applied yet in the Costa Rican agricultural sector. S-LCA studies are still scarce, and most of the time, the assessments focus on few stakeholder groups, as stated by Sharaai and Mokti (2020). Studies in the environmental dimension through LCA are beginning to increase in the LAC Region and Costa Rica; however, when conducting searches on databases like Scopus, the keywords ‘*Life Cycle Assessment Costa Rica*’ provided 16 documents, and not all of them referred to agriculture or food products. In addition, only one paper regarding the inclusion of LCT approaches in tertiary educational contexts in Costa Rica was located. That study suggested there are needed improvements in all the areas of LCT, but particular emphasis was placed on the need to improve the accounting for social implications (Cornejo and Orner 2019).

The selection of a characterisation method was another point of debate for the researchers, and SAM was considered a suitable one for the clearness of results and the possibility of having formal sources to provide the fundament for the scale of values. It also eliminated the constrain of the cost of acquisition of databases, as in other alternatives, but then potential bias could always be present.

A systematic review on the evolution of the S-LCA guidelines by Tokede and Traverso (2020) covered many of the challenges mentioned above; therefore, suggestions included evaluating value chain actors in more consistent ways, with a more robust theoretical orientation for the assessments. Context also has a significant role that needs to be considered in terms of selecting indicators, the need for inclusive and flexible studies and context-oriented choices of functional units. This was evident in sections of the studied cases in our research, where certain indicators would not be applicable in certain cases (indigenous rights for instance), or when the selection of value chain actors rested mostly on farmers.

Finally, the S-LCA method is considered by researchers and sectoral actors keener to understand more about it, as a powerful tool to register the social performance of their sector performance. In addition, it can help trigger improvements in agricultural subsectors or production systems based on the sustainable production and consumption approach. Moreover, the detection of hotspots can help prioritise interventions aligned with local, regional or global policies and goals (Soltanpour et al. 2019; Di Noi et al. 2020) as well as markets and value chains. The step-wise procedures based on the standardised steps for LCA brought into the S-LCA seem to present a path to be followed, and even when several aspects entailed in the UNEP Guidelines are already considered in different certification schemes, the researchers found that the S-LCA basis can provide clearer and systematised suggestions of evidence to respond not only to S-LCA itself but to other schemes as well.

## Conclusions and recommendations

Social assessment requires a deep understanding of both the product or the supply chain to be analysed and the context where the operations take place. Considering the life cycle of products, this could represent different locations and contexts for the production, the consumption and the end of life stages. This condition needs to be observed when interpreting the results from this study which focuses on the primary and early processing operations taking place in a LAC country, to later be delivered at other consumption locations. The LCT approach is known for its usefulness as a tool for business strategies, policy and decision-making processes and is considered a required step for further communication for value chain actors, consumers and stakeholders. Consequently, the delicate consideration of product and context understanding is a must. In this way, observed results should be interpreted not as a limitation for trade and business opportunities, but as highlights of the opportunities of improvement and deserved attention from all value chain actors. Context is not an excuse but is a reality that needs to be accounted for and carefully regarded.

S-LCA allowed a better understanding of the potential social opportunities and vulnerabilities of the agri-food sector, presented through three cases from a LAC developing country, Costa Rica. Cases belonging to the coffee, dairy and vegetable subsectors suggest hotspot can be located mostly within social impact subcategories related to social security, women’s empowerment and documentation processes of the working relations. However, certain limitations remain in using of the S-LCA techniques, such as data collection, metrics and characterisation methods. SAM provided an efficient and clear way to conduct the assessment; however, careful and robust documentation of BRs is required, together with detailed data collection, tools and sample representativeness to assure objective assessments.

The studied cases represents the first documented S-LCA in these agriculture subsectors of Costa Rica, unveiling challenges to the contextual, technical and social-performance output and experiences and the knowledge of S-LCA and LCT in general by institutional and productive actors. Addressing knowledge, awareness and capacity building in LCT in the country entails paramount opportunities to record, track and document social performances of this type of productive activities. Moreover, the outcomes can be used in public policy orientation. Further research where more stakeholders and increased number of interviewees and representative samples of farms are considered would allow more robust assessments that better support decision-making processes derived from hotspot detection and attention.

## Supplementary information

Below is the link to the electronic supplementary material.

Supplementary file1 (PDF 200 KB)

## Data Availability

The datasets generated during and/or analysed during the current study are available from the corresponding author on reasonable request.
