# Supplementary material for: Unveiling the social performance of selected agri-food chains in Costa Rica: the case of green coffee, raw milk and leafy vegetables
Source: Int J Life Cycle Assess. 2021 Sep 9;26(10):2056–71. doi: 10.1007/s11367-021-01964-4 (PMC8428208; doi:10.1007/s11367-021-01964-4)
Supplement: Supplementary file 1 — Supplementary file1 (PDF 200 KB) [file 11367_2021_1964_MOESM1_ESM.pdf]

Social-Life cycle assessment of food supply chains in Costa Rica: opportunities and vulnerabilities

Laura Brenes-Peralta[1], María Fernanda Jiménez-Morales[2], Rooel Campos-Rodríguez[3], Matteo Vittuari[4]
[1,2,3] Agribusiness School, Tecnológico de Costa Rica; [1,4] DISTAL, University of Bologna

S-LCA indicators

Scale based on Subcategory assessment method (SAM)[1]

A	B	C	D
4	3	2	1
proactive, surpasses BRs	complies with BRs	non-compliant with BRS, similar to its context	non-compliant with BRs, even when context does

Note: BR (basic requirements, build upon international and national standards, regulations and conditions)

1

M. D'Eusanio, M. Serreli, A. Zamagni and L. Petti, "Assessment of social dimension of a jar of honey: A methodological outline," *Journal of Cleaner Production Volume 199,* pp. 503-517 https://doi.org/10.1016/j.jclepro.2018.07.157, 2018.

L. Petti, P. K. Sanchez Ramirez, M. Traverso and C. M. Lie Ugaya, "An Italian tomato BCuore di Bue^ case study: challenges and benefits using subcategory assessment method for social life cycle assessment," *International Journal of Life Cycle Assessment Volume 23 ,* pp. 569–580 DOI 10.1007/s11367-016-1175-9, 2018.

P. K. Sanchez Ramirez, L. Petti, N. T. Haberland and C. M. Lie Ugaya, "Subcategory assessment method for social life cycle assessment. Part 1: methodological framework," *International Journal of Life Cycle Assessment Volume 19 ,* pp. 1515–1523 DOI 10.1007/s11367-014-0761-y, 2014.

Stakeholder: Farmer

	name of subcategory	Aim of assessment [2]	indicators
1	Meeting basic needs	To assess the extent to which the basics needs of farmers are met and the extent to which a contribution is made towards improving the status quo.	F1.1. Access to potable water<br>F1.2. Access to wastewater disposal and treatment Service<br>F1.3. Access to electricity service<br>F1.4. Access to enough food (food security)
2	Access to services and inputs	To evaluate the extent to which farmers have access to inputs such as credit, banking or a secure method for storing and saving money, good-quality seeds, and services such as ICT, electricity and infrastructure (e.g. roads, bridges, schools). This social topic aims to assess both local conditions and the contributions made by value-chain actors.	F2.1. Access to telephone Service<br>F2.2. Internet coverage<br>F2.3. Physical access to the farm (accessible roads through the year)<br>F2.4. Presence of nearby public transportation<br>F2.5. Access to production inputs<br>F2.6. Access to supplementary services (extension and training, credit, insurance)
3	Women´s empowerment, inclusion and non-discrimination practices	To assess the extent to which a role of female farmers is recognised within the value chain and the extent to which contributions are made to empower female small-scale farmers (i.e. equal access to jobs, training, advancement and benefits, and other rights for women, as well as opportunities to maintain cultural identity).	F3.1. Gender balance<br>F3.2. Integration of diverse populations<br>F3.3. Presence of women in leading or decision-making roles<br>F3.4. Equal pay for men and women<br>F3.5. Evidence of Empowerment, equity and diversity policies (actual or factual)

[2] Descriptions of the aim of the subcategory assessment for the tables of farmers, workers and local community based or extracted from:

UNEP/SETAC, The Methodological Sheets for Subcategories in Social Life Cycle Ass essm ent (S-LCA), Paris https://www.lifecycleinitiative.org/wp-content/uploads/2013/11/S-LCA_methodological_sheets_11.11.13.pdf, 2013.

M. Goedkoop, D. Indrane and . I. de Beer, Product Social Impact Assessment Methodology Report 2018, Amersfoort: PRé Consultants BV and the Roundtable for Product Social Metrics, 2018.

4	Child Labour	To identify if child labour takes place, since it is defined as work that deprives children of their childhood, their potential and their dignity, and is harmful to physical and mental development. Minor children can work at their own parents' farm, or workshop in activities not considered hazardous, as long as this does not affect their school attendance and their moral, social and physical development. Work must be appropriate to the subject's age and physical condition.	F4.1. Absence of underage workers (<15 years old, according to national legislation in allowed tasks)<br>F4.2. Absence of underage workers (<15<18 years old) in extended working days<br>F4.3. Related or family minors are in formal educational system<br>F4.4. Evidence of Child Labour prevention policies or mechanisms
5	Health and safety	Defined as the extent to which farmers maintains safe working conditions for themselves, their families and workers. This social topic aims to measure the risks associated with farmers working conditions and the extent to which the activity/farm is making contributions to good safety procedures by engaging related actors in training programmes, awareness raising events, etc.	F5.1. Low accendibility /year related to work<br>F5.2. No fatalities related to work<br>F5.3. Evidence of job security and accident prevention policies or mechanisms<br>F5.4. Low rate of incapacities and illnesses/year<br>F5.5. Safety material is provided to workers<br>F5.6. Safety material is used by workers<br>F5.7. Training and capacity to prevent accidents and job security<br>F5.8. Use of signals for delimitation of areas (storage, process, transit, high voltage, etc)<br>F5.9. Access to basic hygiene conditions (water, soap, toilet or restroom)
6	Land rights	To assess the farmers' legal rights to land and tenure security.	F6.1. Production is done in own land<br>F6.2. If rented, basic conditions are supported by contracts, fair rental price, among others.<br>F6.3. No evidence of threads to land rights
7	Corporate responsibility	To assesses to what extent an organization is engaged in reducing its negative impacts that affect sustainability.	OS1.1. Contribution of the activity to national food safety and security<br>OS1.2. Contribution of the activity to national economic development<br>OS1.3. Promotion of compliance with social and labour security<br>OS1.4. Promotion of compliance with health and environment regulations<br>OS1.5. Evidence of Good practices (GAP, GMP, animal welfare)

8	Fair trading relationships	To evidence the quality of the trading relationship of the farmers within the value-chain	OS2.1. Presence of organizations that represent the sector (farmers' association, chamber, etc.)<br>OS2.2. Evidence of inter and intra-sector alliances<br>OS2.3. Local suppliers' preference<br>OS2.4. Presence of regulations and practices promoting fair-trade conditions<br>OS2.5. Sustainable purchases principles are in place

Stakeholder: Worker

	name of subcategory	Aim of assessment	indicators
1	Freedom of association and Collective Bargaining	To verify the compliance of the organization with freedom of association and collective bargaining standards.	W1.1. No evidence that collective bargaining of association is forbidden<br>W1.2. Presence of organized workers' groups<br>W1.3. % of workers that are affiliated to a group, association<br>W1.4. Evidence/testimony of agreements between employees and employers in reference to working conditions
2	Child labour	To verify if the organization might or is employing children (as defined in the ILO conventions) and to identify the nature of any child labour.	W2.1. No presence of underage workers (<15 years old, according to national legislation in allowed tasks)<br>W2.2. No presence of underage workers (<15<18 years old) in extended working days<br>W2.3. Related or family minors are in formal educational system<br>W2.4. Evidence of Child Labour prevention policies or mechanisms
3	Fair salary	To assess whether practices concerning wages are in compliance with established standards and if the wage that is payed meets legal requirements, whether it is above, meeting or below sector average and whether it can be considered as a living wage	W3.1. Salary is equal or above minimum in the country by law.<br>W3.2. The salary allows the worker to fulfil basic needs (food, health, housing)<br>W3.3. Evidence of payments (receipts, payroll records, digital information system)<br>W3.4. Deductions are not applied in arbitrary manners

4	Hours of work	To verify if the number of hours really worked is in accordance with the ILO standards and when overtime occurs, compensation in terms of money or free time is planned and provided to workers.	W 4.1. Weekly worked hours fit national regulation (48 hours/week)<br>W4.2. Weekly extra-worked hours do not surpass national regulation (ordinary and extraordinary won´t surpass 12hrs/day when summed)<br>W4.3. Workers can enjoy a 1 day of rest/week and holidays<br>W4.4. Respect to agreed working Schedule<br>W4.5. Evidence/testimony of communication and consensus mechanisms when extraordinary working schedules are needed
5	Forced Labour	To verify that there is no use of forced or compulsory labour in the organization	W5.1. Workers come to work freely<br>W5.2. Contract conditions are clear for the worker<br>W5.3. Evidence or register of working contract (written or oral when applicable)<br>W5.4. Personal documents from workers are never retained<br>W5.5. Workers can quit freely, with corresponding notice when applicable
6	Equal opportunities/ no-Discrimination	To assess equal opportunity management practices and the presence of discrimination in the opportunities offer to the workers by the organizations and in the working conditions.	W6.1. Gender balance within delegated decision-makers or working structure<br>W6.2. Gender balance in the general working structure (peers)<br>W6.3. Integration of diverse populations<br>W6.4. No reported incidents related to discrimination<br>W6.5. The worker states not having felt discriminated<br>W6.6. Evidence of non-discrimination policies (actual or factual) and practices<br>W6.7. Absence of illegal workers<br>W6.8. Evidence of opportunities for training, capacity biulding and education inside or outside the organization.
7	Health and security	To assess both the rate of incidents and the status of prevention measure and management practices. An incident is defined as a work-related event(s) in which a injury or ill health (regardless of severity) or fatality occurred or could have occurred.	W7.1. Low accendibility /year related to work<br>W7.2. No fatalities related to work<br>W7.3. Evidence of job security and accident prevention policies or mechanisms<br>W7.4. Low rate of incapacities and illnesses/year<br>W7.5. Safety material is provided to workers<br>W7.6. Safety material is used by workers

			W7.7. Training and capacity to prevent accidents and job security<br>W7.8. Use of signals for delimitation of areas (storage, process, transit, high voltage, etc)<br>W7.9. Access to basic hygiene conditions (water, soap, toilet or restroom)
8	Social Benefits/Social Security	To assess whether an organization provides for social benefits and social security of workers and to what extent.	W8.1. Worker is covered by minimum social security payed by employer<br>W8.2. Worker is covered by job- risks security<br>W8.3. absence of incidents related to employers' social security breach<br>W8.4. Access to resting areas<br>W8.5. Promotion of healthy habits and lifestyle<br>W8.6. Flexibility in working hours and conditions (extraordinary permits, etc.)

Stakeholder: Local Community

number	name of subcategory	Aim and approach of indicator assessment	indicators
1	Delocalization and migration	The assessment aims to assess whether organizations contribute to delocalization, migration or "involuntary resettlement" within communities and whether populations are treated adequately.	LC1.1. Absence of emigration due to the farm activity<br>LC1.2. Immigration attracted due to decent job opportunities created by the farm<br>LC1.3. Immigrants integrate to the community they arrived at<br>LC1.4. Evidence or policies or mechanisms supporting better and decent conditions for immigrants
2	Community involvement	This subcategory assesses whether an organization includes community stakeholders in relevant decision-making processes. It also considers the extent to which the organization engages with the community, in general.	LC2.1. Involvement of the farm (farmer, family, employees) in local activities to promote development, awareness, volunteering, in environmental, health, emergency aspects).<br>LC2.2. Involvement in Local Development Associations (municipality) or boards<br>LC2.3. Existence of Alternate conflict resolution mechanisms

3	Cultural heritage	This subcategory assesses whether an organization respects local cultural heritage and recognizes that all community members have a right to pursue their cultural development	LC3.1. Evidence of inclusion and respect for cultural heritage within productive practices<br>LC3.2. Involvement in activities related to the rescue of cultural heritage<br>LC3.3. Translation of related productive/farm information into local native language
4	Respect of Indigenous Rights	This subcategory assesses organizational respect for the rights of indigenous peoples, as a group or as individuals.	LC4.1. Existence of policies protecting Indigenous Rights<br>LC4.2. Indigenous peoples' integration to local community and activities<br>LC4.3. Existence of sessions or specific groups supporting indigenous rights or consensus in decision -making processes.<br>LC4.4. Absence of incidents with Indigenous peoples' (discrimination, disrespect, etc)
5	Local Employment	This subcategory assesses the role of an organization in directly or indirectly affecting local employment	LC5.1. Evident local employment<br>LC5.2. Evidence of policies (actual or factual) or mechanisms for preference of local employees<br>LC5.3. Preference for local provisioning
6	Access to Immaterial Resources	This subcategory assesses the extent to which organizations respect, work to protect, to provide or to improve community access to immaterial resources.	LC6.1. Involvement in educational activities in the community<br>LC6.2. Low incidence of conflicts with the community
7	Access to material resources	This subcategory assesses the extent to which organizations respect, work to protect, to provide or to improve community access to local material resources (i.e. water, land, mineral and biological resources) and infrastructure (i.e. roads, sanitation facilities, schools, etc.).	LC7.1. Support to the community in tangible resources improvement (roads, electrification, buildings, related services)<br>LC7.2. The farm activity represent low risk to tangible resources of the community, such as water, soil, other resources<br>LC7.3. Evidence of environmental management systems or responsible practices
8	Safe and Healthy Living Conditions	This subcategory assesses how organizations impact community safety and health. This includes the general safety conditions of operations and their public health impacts.	LC8.1. Absence of environmental or physical incidents related to the farm (pollution Events, damage of infrastructure)<br>LC8.2. Absence of health-related incidents due to the farm<br>LC8.3. Support for improved security and health local conditions

9	Secure Living Conditions	This subcategory assesses how organizations impact the security of local communities with respect to the conduct of private security personnel and how the organization interacts with state-led forces.	LC9.1. Evidence of policies related to responsible security personnel practices (weapon possession and permits, training).<br>LC9.2. Absence of incidents related to security personnel of the farm (shootings, abuse of authority, violence)<br>LC9.3. Absence of fatalities related to security personnel of the farm
